# A Catalogue of the Pathological Cabinet of the New York Hospital

**Published:** 1861-03

**Authors:** 


ART. VI.—*A Catalogue of the Pathological Cabinet of the New York Hospital, classified and arranged.* By ROBERT RAY, JR., M.D. 8vo., pp. 364. New York, 1860.

THIS work is a model of its kind, reflecting great credit upon its author. The collection which it describes is divided into eight sections: I. Bones. II. Joints and tendons. III. Digestive system. IV. Respiratory system. V. Circulatory system. VI. Nervous system and organs of the senses. VII. Genital and urinary system. VIII. Parasites. It embraces nearly one thousand specimens, put up in admirable style, well labeled, and fully described in the excellent catalogue before us, thus rendering reference at once easy and instructive. The value of such a collection can be properly appreciated only by those who are thirsting after a knowledge of morbid anatomy. New York has, indeed, much reason to be proud of it. We know of no similar collection of equal extent anywhere in the country.

The first steps toward its formation were taken in 1840, at the instance of Dr. John Watson, whose disinterested zeal in the cause of medical science, and whose great learning as a surgeon and a physician, reflect so much credit upon the profession of New York.

The catalogue is prefaced by a brief memoir of the author, the late Curator of the Museum, by his friend and early preceptor, Dr. Watson. From this we learn that Dr. Ray was born in the City of New York in 1832, that he was a zealous and enthusiastic student, that he received his medical degree at the College of Physicians and Surgeons in 1856, that he was one of the house surgeons of the New York Hospital, and subsequently a Dispensary physician, and that he died in July, 1860, meeting his fate with the composure and resignation of a Christian.

"Dr. Ray," says his biographer, "had but little taste for the gayeties of society. He was rarely seen at places of fashionable resort. His hours of relaxation were mostly devoted to the family circle, or spent in friendly intercourse with a few familiar acquaintances. He had a cultivated taste for music, and performed with skill on the piano and violin."

